# Poorer Exercise Accommodation of Regional Systolic Myocardial Motion after Spironolactone Treatment in Heart Failure Patients with Preserved Ejection Fraction and Ventricular Dyssynchrony

**DOI:** 10.3390/jcm10173827

**Published:** 2021-08-26

**Authors:** Chih-Chieh Yu, Fu-Chun Chiu, Chia-Ti Tsai, Yi-Chih Wang, Ling-Ping Lai, Juey-Jen Hwang, Jiunn-Lee Lin

**Affiliations:** 1 Department of Internal Medicine, National Taiwan University Hospital, No. 7, Chung-San South Road, Taipei 100, Taiwan; sweetchieh@gmail.com (C.-C.Y.); fang31@ms39.hinet.net (C.-T.T.); lplai2003@ntu.edu.tw (L.-P.L.); jueyhwang@ntu.edu.tw (J.-J.H.); jiunnlee@ntu.edu.tw (J.-L.L.); 2 Department of Internal Medicine, National Taiwan University Hospital, Yun-Lin Branch, Yunlin 640203, Taiwan; uenling@hotmail.com

**Keywords:** heart failure, aldosterone antagonism, dyssynchrony, myocardial motion, exercise

## Abstract

Patients with heart failure and preserved ejection fraction (HFpEF) are known to have reduced systolic myocardial velocity (Sm) with impaired accommodation to exercise. We tested the impact of an aldosterone antagonist on Sm at rest and post-exercise. Forty-nine HFpEF patients (65 ± 11 years, 24 male) with HF signs/symptoms, mitral E/Ea (annular early diastolic velocity) > 8, and left ventricular (LV) EF > 50% were randomized to spironolactone (25 mg/day, 25 patients) or the Control. At baseline and 6 months, we analyzed Sm of basal LV segments at rest and after a 6 min treadmill exercise. At 6 months, post-exercise mean Sm in the spironolactone group became greater than that in the Control (9.2 ± 1.6 vs. 8.3 ± 1.0 cm/s, *p* = 0.021), mainly due to the increment of post-exercise % increase of lateral Sm (44 ± 30 vs. 30 ± 19% at baseline, *p* = 0.045). Further analyses showed the presence of systolic dyssynchrony (standard deviation of electromechanical delay of 6-basal LV segments > 35 ms) was independently associated with a poorer response to spironolactone, defined as a post-exercise % increase of lateral Sm < 50% (OR = 2.7, 95% CI = 1.8–4.2) and the increment of Ea < 1.5 cm/s (OR = 1.5, 95% CI = 1.1–2.3). Spironolactone could improve exercise accommodation of regional systolic myocardial velocity for HFpEF patients. However, its benefits could be decreased in those with ventricular dyssynchrony. This suggested possible therapeutic impacts from underlying heterogeneity within HFpEF patients.

## 1. Introduction

Patients with heart failure and preserved left ventricular (LV) ejection fraction (HFpEF) account for nearly half of all of the HF population. Diastolic dysfunction has been considered as the core pathophysiology for the development of HFpEF. As well as impaired diastolic function, reduced systolic motion of the longitudinal axis detected by tissue-Doppler imaging (TDI) has been identified in patients with HFpEF in several studies [1,2,3]. Tan et al. [4] showed that the lower resting mitral annular systolic velocity in patients with HFpEF failed to rise normally on exercise as compared with that of the healthy controls.

Based on the activation of renin-angiotensin-aldosterone system in long-standing hypertension leading to HFpEF, aldosterone antagonism has been suggested to provide potential therapeutic benefits for HFpEF patients with the improvement of diastolic function in the Randomized Aldosterone Antagonism in Heart Failure with Preserved Ejection Fraction Trial (RAAM-PEF) [5] and the Aldo-DHF trial [6]. However, with the paucity of studies evaluating whether aldosterone antagonism could improve systolic characteristics in HFpEF patients, we conducted the study to test the efficacy of aldosterone antagonism on rest and post-exercise adaptation of myocardial systolic motion.

Furthermore, when evaluating post-exercise systolic myocardial motion in HFpEF patients, the possible detrimental role of underlying systolic ventricular dyssynchrony derived by TDI should be considered. Our earlier study demonstrated that the presence of systolic dyssynchrony in HFpEF patients was associated with the significantly poorer adaptation of mean systolic myocardial motion to exercise (6.6 ± 0.9 to 7.9 ± 1.3 cm/s) when compared with those without dyssynchrony (6.8 ± 1.0 to 8.6 ± 1.5 cm/s) [7]. Therefore, we further compared the pharmacological efficacy of aldosterone antagonism on HFpEF patients based on the presence or absence of ventricular dyssynchrony in the spironolactone arm.

## 2. Methods

### 2.1. Study Design and Population

We performed a single-center, prospective, randomized, and open-label trial to investigate the impacts of spironolactone on myocardial systolic motion in hypertensive patients with HFpEF (ClinicalTrials.gov number NCT01944384).

Hypertensive patients were diagnosed to have HFpEF if they were presenting exertional dyspnea (New York Heart Association functional class II/III) or HF signs/symptoms meeting the Framingham criteria, mitral E-flow/annular early diastolic velocity (E/Ea) > 8, and LVEF > 50% [7,8]. All HFpEF patients were randomized in a 1:1 ratio to the Spironolactone (25 mg per day) group or the Control group for a period of 6 months. The randomization process was according to the last number of their chart number in the study hospital. Patients were randomized to the Control if the last number was even, and to the Spironolactone group if it was odd. Before randomization, patients should have stabilized blood pressure (<140/90 mmHg) and signs/symptoms of HF for at least 3 months according to institutional medical records. Otherwise, factors such as the occurrence of paroxysmal atrial fibrillation, or active ischemia which were relating to the signs or symptoms of HF should also be corrected or stabilized by either medications or interventions for more than three months before the evaluation of echocardiography and randomization.

Due to the study design, patients who could not tolerate the exercise test were excluded. Other exclusions included baseline blood pressure <100/60 mmHg, secondary hypertension, restrictive, constrictive, or hypertrophic cardiomyopathy, more than moderate valvular heart diseases, chronic atrial fibrillation, usage of aldosterone antagonism in the past, chronic pulmonary disease, acute coronary syndrome within 3 months, positive cardiac stress tests, untreated known stenoses >50% in major coronary arteries, baseline serum potassium ≥ 5.0 mEq/L, or serum creatinine concentration more than 2.0 mg/dL. The study protocol was approved by the institutional ethics committee (201701059MINC) of the National Taiwan University Hospital on 13 June 2017, and written informed consents were obtained from the patients.

### 2.2. Follow-Up and Safety Monitoring

Serum potassium was monitored at 1 and 3 months after randomization, and then at the end of the study. Spironolactone was necessarily discontinued in the presence of significant hyperkalemia (serum potassium > 5.5 mEq/L) or other side effects intolerable to the patient. The other cardiovascular medications were kept unchanged during the study period.

### 2.3. Echocardiography

All patients received standard echocardiography coupled with TDI (iE33, Philips; Andover, MA) with a 1- to 5-MHz transducer at baseline and six months after randomization. Chamber sizes, LVEF, left atrial volume index (LAVI), and LV mass (g) (LVM) were measured and calculated. LVM was indexed to height to the power of 2.7 (LVM/Ht^2.7^) [9]. Isovolumic relaxation time (IVRT) was measured as the time interval between the end of LV outflow and the start of LV inflow signals using a continuous-wave beam directed from the apical five-chamber view. In TDI, mitral Ea was the mean of septal and lateral mitral annular early diastolic velocities. TDI of the 6-basal LV segments including septal, anteroseptal, anterior, lateral, posterior, and inferior aspects were studied using apical views for the long-axis motion of the ventricle. A frame rate pulsed Doppler scanning of 120 Hz was used. In this way, the peak systolic myocardial velocity (Sm) during the ejection phase of each segment was measured, and the absolute time difference of electromechanical delay from QRS onset to peak Sm (Ts) between the septum and each segment was calculated [7,10]. The presence of ventricular dyssynchrony was the standard deviation of Ts among 6-basal LV segments (Ts-SD) > 35 msec [10,11]. All measurements were analyzed offline. The echocardiography was performed by a cardiologist blinded to the patient’s condition. The intra-observer variability was 1.49% for mitral E-flow, 1.67% for mitral Ea, 1.09% for mean Sm, and 1.91% for Ts. The corresponding values for interobserver variability were 4.55%, 2.38%, 2.38%, and 3.84% [7].

### 2.4. Exercise Protocol

After echocardiography evaluation at rest, all patients completed a treadmill exercise test (Exercise System CH 2000, Cambridge Heart, Inc., Wilmington, MA, USA) for up to 6 min using the Bruce protocol (stage 2: 2.5 MPH, slope = 12%, 7.05 Mets). Then, patients were lying on the couch beside the treadmill machine, and post-exercise TDI recordings were immediately performed [7,10].

### 2.5. Quality of Life Evaluation

Quality-of-life (QOL) score was assessed at baseline and at six-months by using the Minnesota Living with Heart Failure questionnaire (Chinese version).

### 2.6. Laboratory Measurement

Blood samples for quantifying N-terminal pro-brain natriuretic peptide (NT-proBNP) were drawn from all patients at baseline and at 6 months. The plasma was frozen at −80 °C until the immunoassay (Elecsys ProBNP, Roche Diagnostics, Indianapolis, IN, USA).

### 2.7. Statistical Analysis

With referenced data of our earlier studies [7,10], we assumed an important change in post-exercise mean Sm of 0.7 cm/s from baseline to 6 months, a standard deviation of post-exercise mean Sm of 1.0 cm/s, and a 2-sided α = 0.05; a sample size of 43 patients was estimated, which provided 90% power for the study end point. The distributional properties of continuous variables were expressed by mean ± standard deviation (SD) and categorical variables were presented by frequency and percentage. To evaluate the effect of spironolactone, parameters at baseline and at 6 months were compared by paired *t*-test. The % increase was calculated as the formula: (post-exercise value − baseline value)/(baseline value) × 100. In univariate analysis, the differences in the distributions of continuous variables and categorical variables were examined using Wilcoxon rank-sum test and Chi-square test as appropriate for the data type. In multivariate logistic regression analysis, a forward stepwise model was used. All analyses were performed using the SPSS 17.0 software package (SPSS Inc., Chicago, IL, USA).

## 3. Results

### 3.1. Baseline Characteristics

After screening 90 HFpEF patients, 41 patients were excluded due to the exclusion criteria (12 patients could not tolerate the exercise test; 2 with significant valvular heart disease; 2 with hypertrophic cardiomyopathy; 2 with arrhythmias potentially confounding the results; 20 were angina-free but with positive stress tests) or declination to adhere to the protocol (three patients). Finally, 49 HFpEF patients (65 ± 11 years, 24 male) were randomized. Among them, 25 patients were the Spironolactone group, and the other 24 patients were the Control group (Table 1). All patients completed the follow-up period without specific adverse events.

At baseline, the Control and Spironolactone groups were similar in age, gender, and other clinical features. The baseline blood pressures, QOL scores, potassium and NT-proBNP levels, and echocardiographic parameters were also similar between the two groups.

### 3.2. Comparisons at Follow-Up

At 6 months, the blood pressure did not change significantly when compared to that at baseline, and remained similar between the two groups (Table 2). The Spironolactone group had a significantly higher potassium level at 6 months, when compared with that at baseline (*p* = 0.011 by paired *t*-test) and that in the Control group. However, no patients in the spironolactone group developed a potassium level >5.5 mEq/L. Two patients (8%) reported tolerable nipple pain in the spironolactone arm, compared with 0% in the Control. No gynecomastia was found in the spironolactone group. The QOL score of the Spironolactone group was getting better at follow-up (*p* = 0.018 versus baseline by paired *t*-test), but it did not reach a statistical difference from that of the Control group.

In echocardiography, the chamber sizes and wall thickness at follow-up in the Spironolactone group neither differed from the Control group, nor changed significantly when compared to the baseline values. Among diastolic parameters, there was significant improvement in mitral Ea, E/A, and E/Ea at follow up (*p* < 0.05 versus baseline by paired *t*-test) in the Spironolactone group. However, the diastolic parameters also did not reach significant differences between the Spironolactone and Control group at 6 months.

### 3.3. Comparisons of Systolic Motion at Baseline and Follow-Up

The mean Sm of the 6-basal myocardial segments were similar at baseline and at follow-up between the two groups (Table 3). After exercise provocation, however, the post-exercise mean Sm in the Spironolactone group became significantly greater than that in the Control group at 6 months. The increase of post-exercise mean Sm in the Spironolactone group was mainly due to a much greater post-exercise % increase of lateral Sm than that of the Control group.

### 3.4. Impacts of Ventricular Dyssynchrony on Post-Exercise Accommodation of Systolic Myocardial Velocity after Spironolactone Treatment

Among 25 patients randomized to spironolactone treatment, we identified 13 patients with ventricular dyssynchrony (Ts-SD: 56.1 ± 12.1 ms) at baseline as the Dys subgroup, and the others were the Non-dys subgroup (Ts-SD: 13.6 ± 9.5 ms). Patients in the Dys subgroup were older (71 ± 7 vs. 61 ± 9 years, *p* = 0.004) with more prescription of diuretics (92 vs. 25%, *p* < 0.001) but less use of β-blockers (69 vs. 100%, *p* = 0.037) than the Non-dys subgroup. They also had a borderline lower mitra Ea when compared with the Non-dys subgroup (*p* = 0.05). The other clinical and echo-parameters at baseline were similar between the two subgroups.

**Figure 1:**
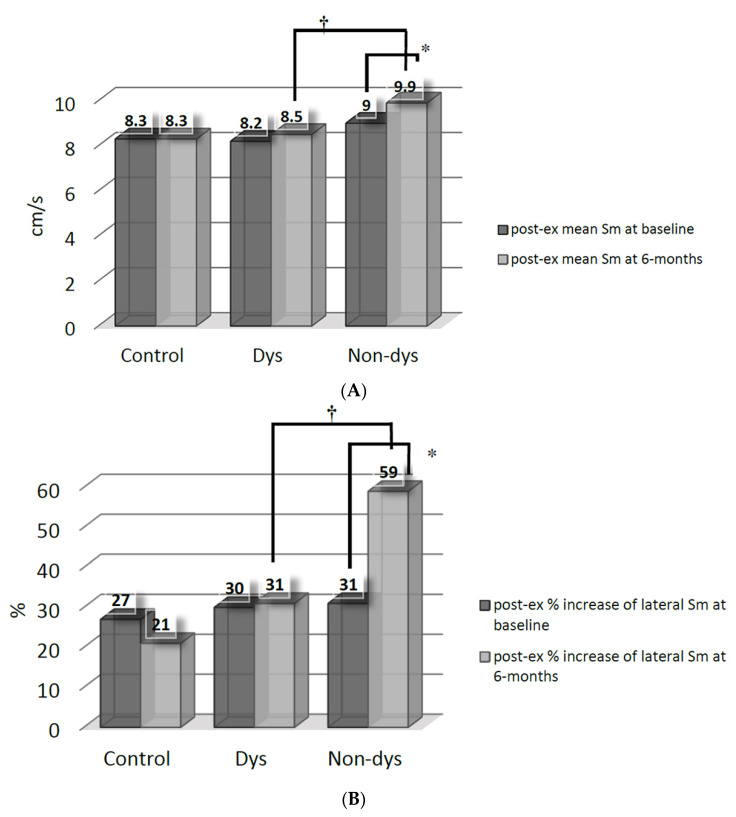
(**A**) Comparisons of post-exercise (post-ex) mean systolic myocardial velocity (Sm) between the dyssynchrony (Dys) and non-dyssynchrony (Non-dys) groups at baseline and at 6 months after spironolactone therapy. * *p* < 0.05 versus baseline; † *p* < 0.05 between the Dys and Non-dys subgroups. (B) Comparisons of post-exercise (post-ex) % increase of systolic myocardial velocity (Sm) between dyssynchrony (Dys) and non-dyssynchrony (Non-dys) group at basal-lateral segment at baseline and at 6 months after spironolactone therapy. * *p* < 0.05 versus baseline; † *p* < 0.05 between Dys and Non-dys subgroup.

After spironolactone therapy, the increment (*p* < 0.05 versus baseline by paired *t*-test) of post-exercise mean Sm (Figure 1A) and % increase of lateral Sm (Figure 1B) were only significant in the Non-dys subgroup. The Non-dys subgroup, therefore, had a greater post-exercise mean Sm and % increase of lateral Sm than the Dys subgroup at 6 months.

**Figure 2:**
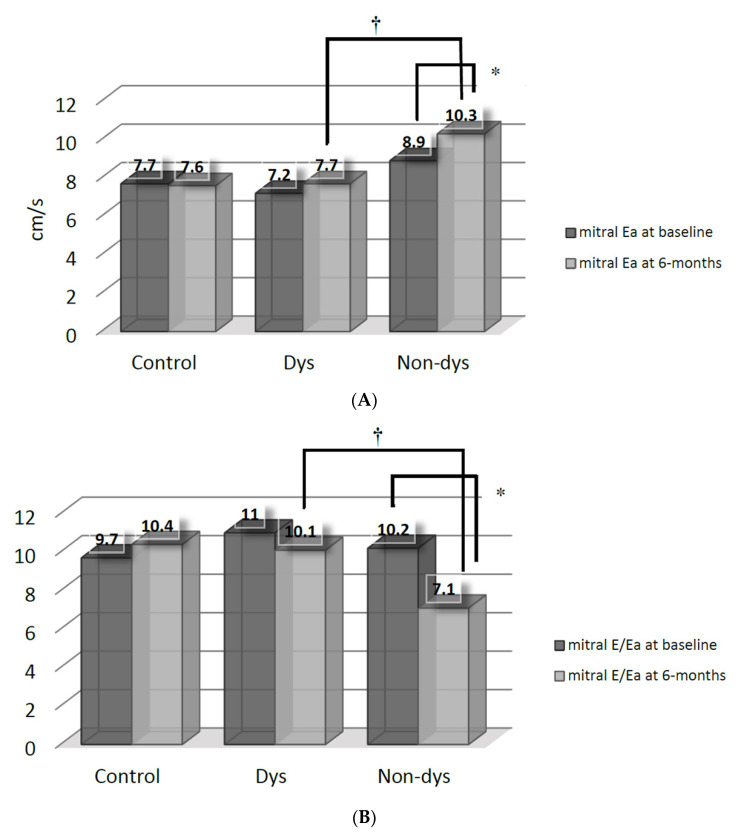
(**A**) Comparisons of mitral annular early diastolic velocity (Ea) between the dyssynchrony (Dys) and non-dyssynchrony (Non-dys) groups at baseline and at 6 months after spironolactone therapy. * *p* < 0.05 versus baseline; † *p* < 0.05 between the Dys and Non-dys subgroups. (**B**) Comparisons of mitral E-flow/annular early diastolic velocity (Ea) between the dyssynchrony (Dys) and non-dyssynchrony (Non-dys) groups at baseline and at 6 months after spironolactone therapy. * *p* < 0.05 versus baseline; † *p* < 0.05 between the Dys and Non-dys subgroups.

The improvements (*p* < 0.05 versus baseline by paired *t*-test) of mitral Ea (Figure 2A) and E/Ea (Figure 2B) were also mainly found in the Non-dys, but not in the Dys subgroup. Consequently, the Non-dys subroup had higher mitral Ea and lower E/Ea, when compared with the Dys subgroup at 6 months.

With the reference of our previous study [7], we arbitrarily chose the post-exercise % increase of lateral Sm ≥50% and increase of mitral Ea ≥1.5 cm/s as good responses to spironolactone therapy at 6 months. In multivariate analyses after correction for age, gender, cardiovascular medications, mitral E/A, and mitral deceleration time, the presence of ventricular dyssynchrony in HFpEF patients was independently associated with the post-exercise % increase of lateral Sm <50% (odds ratio = 2.7, 95% confidence interval = 1.8–4.2, *p* < 0.001). A poorer increment of mitral Ea (<1.5 cm/s: odds ratio = 1.5, 95% confidence interval = 1.1–2.3, *p* = 0.028) in the Dys subgroup was also found among HFpEF patients receiving spironolactone therapy.

## 4. Discussion

This study showed that a much higher increase of post-exercise mean Sm, mainly due to the increment of lateral Sm after exercise, was found in HFpEF patients with spironolactone treatment. Further analysis suggested that HFpEF patients without ventricular dyssynchrony could have a better response to spironolactone with respect to both post-exercise % increase of lateral Sm and mitral Ea. These findings raised the concern that the presence of ventricular dyssynchrony, as possible underlying heterogeneity within HFpEF patients, could interfere with the overall efficacy of spironolactone therapy.

Unlike diastolic function, previous HFpEF trials evaluating the response of myocardial systolic behavior to pharmacological therapy were rare. Mottram et al. [12] randomized 30 hypertensive patients with exertional dyspnea and diastolic dysfunction to receive spironolactone or placebo. At 6 months, there were neither significant differences in mitral septal (6.5 vs. placebo: 6.2 cm/s) and lateral (7.2 vs. placebo: 7.0 cm/s) annular systolic velocities between the two groups, nor significant changes when comparing with the baseline values in spironolactone group (septal: 6.5 vs. baseline 6.1 cm/s; lateral: 7.2 vs. baseline 7.4 cm/s). The results were comparable to the findings of the current study, which did not show any significant difference in mean resting Sm between the spironolactone and the control group (7.2 vs. control: 7.1 cm/s) at 6 months. However, by further evaluating post-exercise accommodation of systolic myocardial velocity, we demonstrated a significant improvement of post-exercise mean Sm in HFpEF patients receiving spironolactone treatment. Tan’s group [4] showed that reduced systolic mitral annular velocity at rest in HFpEF patients failed to rise after exercise as normally as that in healthy controls. They also found that the post-exercise systolic annular velocity correlated positively with maximum oxygen consumption (VO_2_max, r = 0.61, *p* = 0.003), and therefore suggested that reduced increment of systolic annular velocity after exercise could contribute partially to exercise limitation in HFpEF patients. Based on that study, the increased post-exercise systolic myocardial velocity after spironolactone therapy for 6 months shown in the current study could potentially translate into improved exercise tolerance for HFpEF patients.

When further analyzing if the presence of ventricular dyssynchrony would alter pharmacological response in the spironolactone group, we found that HFpEF patients with dyssynchrony were unable to achieve significant improvement with respect to both post-exercise adaptation of systolic myocardial motion and diastolic parameters. In the literature, the presence of systolic dyssynchrony was estimated to be in 17–39% of HFpEF patients [13,14,15]. However, the clinical implication of mechanical dyssynchrony for HFpEF patients remains largely uncertain. Similar to patients with HF with reduced EF, HFpEF patients with ventricular dyssynchrony were found to have more impaired diastolic and systolic function than those without dyssynchrony [7,14]. In our earlier study [7], we also demonstrated that the presence of electromechanical delay could compromise exercise accommodation of systolic myocardial motionin HFpEF patients. Basic studies showed that the more delayed-activated ventricular lateral segment would encounter a much greater hemodynamic load than the septum, resulting in lower expression of key proteins involved in muscle mechanics over the LV free wall [16,17]. This could possibly lead to the differential pharmacological responses mainly occurring in the lateral myocardium shown in the present study.

As the fundamental pathophysiology, improvement of diastolic dysfunction after specific pharmacological treatment remains to be one of the major concerns in HFpEF trials. With a similar patient number as the current study, the RAAM-PEF trial showed a borderline improved mitral Ea (7.6 to 8.4 cm/s, *p* = 0.08) and E/Ea (12.7 to 10.9, *p* = 0.06) after eplerenone treatment for 6 months. When compared to the placebo group, a significant difference was only seen in mitral E/Ea (10.9 vs. 14.4 in placebo, *p* = 0.01), but not in Ea (8.4 vs. 7.1 cm/s in placebo, *p* = 0.12) [5]. The much larger Aldo-DHF trial enrolling 422 HF*p*EF patients demonstrated slight but significant changes in mitral Ea (5.9 to 6.16 cm/s) and E/Ea (12.7 to 12.1) in the spironolactone arm [6]. Our study showed similar, but not consistent, results to the previous reports. Although the mitral Ea and E/Ea in our study were found to improve significantly in spironolactone group after treatment for 6 months, there remained to be no significant difference in diastolic parameters between the spironolactone and the control group. The possible reasons for the inconsistency of diastolic function improvement could be the overall number of patients enrolled, and the potential heterogeneous burden of myocardial ischemia, or other underlying heterogeneity within the HFpEF population. Since diastolic function is rather more susceptible to ischemia than gross systolic function [18], the relationship between the presence of coronary artery disease and diastolic dysfunction despite normal LVEF has been well known [19]. Though the ischemic burden has been minimized in studies investigating HFpEF, the impacts of ischemia still could not be easily excluded. It is caused by the incomplete recovery of diastolic function after revascularization, and the remaining ischemia due to non-revascularized small vessels or microvasculature. The relatively strict exclusion of those with positive stress tests and the lower proportion of patients with coronary artery disease in the present study (20%) than the Aldo-DHF trial (43%) could partially contribute to the different results in the degree of improvement in diastolic function after spironolactone. Up to now, we still cannot have promising long-term outcomes for HFpEF patients from large pharmacological trials including the TOPCAT trial [20] and the recently published PARAGON-HF trial [21]. Since diastolic dysfunction serves as an independent prognosticator even in the general population [22], our study implied the possibility that underlying heterogeneity, such as the presence of dyssynchrony within HFpEF patients, could proportionately alter the overall echocardiographic and even outcome results of these trials.

There were several limitations of the study. First, this was a small randomized, but not placebo-controlled study. Therefore, we did not make emphasis on the comparisons of QOL score due to its subjectivity, especially in such a small study. However, the results of the study were still comparable to other similar trials, and the sensitive changes of systolic motion detected by TDI made the case number enough to achieve a significant result. Second, due to the purpose and exclusion criteria of the study, those who were unable to tolerate the exercise protocol (N = 12) or had myocardial ischemia in stress tests (*n* = 20) were excluded. These population could be the worse group of HFpEF patients. In addition, patients enrolled in the study were required to have stabilized blood pressure and HF signs/symptoms for at least three months. This could contribute to their mildly elevated NT-proBNP and mildly increased E/Ea at baseline. Therefore, these highly selected and treated patients included in this trial could not be regarded as overall HFpEF patients, and whose response to spironolactone could not be completely answered. Third, we did not evaluate the long-term outcomes, so whether the TDI-derived improvement of systolic motion by spironolactone would translate into improved prognosis needs further study. Fourth, angiotensin converting enzyme inhibitors or angiotensin receptor blockers were relatively less used (20%) for patients in the present study, and its interaction with spironolactone is uncertain. Nevertheless, blood pressure stabilization with any kind of anti-hypertensive agent and the use of diuretics for symptom relief were still the class I recommendations for management of HFpEF patients [8]. Fifth, whether there would be further change in systolic or diastolic function beyond 6 months could not be answered in the study. According to the Aldo-DHF trial, there was no additional change of mitral E/Ea but a significant decrease in the LVM index between 6 and 12 months with spironolactone therapy. The contribution of the decreased LVM index to regional myocardial function beyond one year deserves a longer-term study. Sixth, the percentage of the presence of ventricular dyssynchrony was 52% in the spironolactone arm of the study, which was higher than that in the literature [13,14,15]. This made further investigation of the impacts of ventricular dyssynchrony on post-exercise systolic myocardial motion feasible. Truly, the study results could be largely altered if the proportion of ventricular dyssynchrony was extremely low or high enrolled in the spironolactone arm. Nevertheless, it also suggested the possible issue that the underlying heterogeneity might interfere with the results of pharmacological studies for HFpEF patients. Seventh, we did not measure global longitudinal strain derived by speckle tracking echocardiography in the study. This could be a more accurate alternative method to minimize the confounding effects of hyperventilation and hyperdynamic heart after exercise. However, there were several studies investigating post-exercise echocardiographic accommodation by measuring systolic myocardial or mitral annular velocities, as those referenced in this study [4,7,10].

## 5. Conclusions

Prescription of spironolactone for hypertensive patients with HFpEF was associated with improved post-exercise systolic myocardial motion. However, the presence of ventricular dyssynchrony could serve as an independent factor predicting a poorer response to aldosterone antagonism with respect to systolic and diastolic function in these patients. This suggested that the potential interference by underlying heterogeneity within HFpEF patients should be cautiously considered in pharmacological trials.

## Figures and Tables

**Table 1:** Baseline clinical characteristics.

	Control (*n* = 24)	Spironolactone (*n* = 25)	*p*-Value
Age (years)	64 ± 12	66 ± 10	0.540
Gender (male/female)	10/14	14/11	0.326
Body mass index (kg/m^2^)	26.0 ± 3.2	26.0 ± 3.3	0.965
Smoking (*n*)	1	0	0.289
Diabetes (*n*)	6	4	0.445
Hyperlipidemia (*n*)	10	8	0.493
CAD (*n*)	3	5	0.488
History of AF (*n*)	3	6	0.431
NYHA class II/III (*n*/*n*)	16/8	14/11	0.454
Aspirin (*n*)	4	6	0.534
CCB (*n*)	16	21	0.165
β-blocker (*n*)	20	21	0.951
ACEI/ARB (*n*)	7	3	0.142
Diuretics (*n*)	14	15	0.908
Digitalis (*n*)	0	0	
AAA (*n*)	2	5	0.252
Statins (*n*)	5	6	0.796
QRS duration (ms)	88 ± 8	88 ± 8	0.982

AAA, antiarrhythmic agent; ACEI/ARB, angiotensin converting enzyme inhibitor/angiotensin II receptor blocker; AF, atrial fibrillation; CAD, coronary artery disease; CCB, calcium channel blocker; NYHA, New York Heart Association.

**Table 2:** Comparisons at baseline and 6 months.

		Control (*n* = 24)	Spironolactone (*n* = 25)	*p*-Value
Systolic BP (mmHg)	Baseline	131 ± 6	131 ± 6	0.965
	6 months	131 ± 6	129 ± 5	0.191
Diastolic BP (mmHg)	Baseline	77 ± 7	77 ± 5	0.980
	6 months	76 ± 6	77 ± 5	0.807
Potassium (mmol/L)	Baseline	4.1 ± 0.3	4.3 ± 0.4	0.213
	6 months	4.3 ± 0.4	4.5 ± 0.4 *	0.010
QOL score	Baseline	27 ± 22	30 ± 20	0.623
	6 months	24 ± 22	22 ± 20 *	0.818
NT-proBNP (pg/mL)	Baseline	229 ± 281	337 ± 653	0.459
	6 months	182 ± 173	183 ± 213	0.994
LA diameter (mm)	Baseline	37 ± 6	36 ± 5	0.498
	6 months	37 ± 5	36 ± 4	0.212
LAVI (ml/m^2^)	Baseline	24.8 ± 9.2	23.1 ± 9.5	0.548
	6 months	22.9 ± 6.1	21.6 ± 6.1	0.472
IVS (mm)	Baseline	11.3 ± 1.8	11.5 ± 1.9	0.682
	6 months	11.9 ± 1.7	11.9 ± 1.3	0.953
PW (mm)	Baseline	11.4 ± 1.9	11.7 ± 1.9	0.564
	6 months	11.5 ± 1.4	11.1 ± 1.3	0.323
LVEDD (mm)	Baseline	44.5 ± 5.0	46.6 ± 4.1	0.116
	6 months	43.9 ± 5.3	45.0 ± 4.5	0.445
LVESD (mm)	Baseline	28.2 ± 4.6	29.7 ± 3.6	0.223
	6 months	28.0 ± 4.2	28.3 ± 3.8	0.776
RWT	Baseline	0.52 ± 0.10	0.50 ± 0.08	0.519
	6 months	0.54 ± 0.07	0.52 ± 0.05	0.349
LVM/Ht^2.7^ (g/m^2.7^)	Baseline	60.5 ± 17.5	65.9 ± 20.1	0.317
	6 months	62.9 ± 19.2	62.6 ± 14.2	0.944
LVEF (%)	Baseline	67 ± 7	66 ± 6	0.542
	6 months	66 ± 5	67 ± 7	0.684
Mitral E/A	Baseline	0.99 ± 0.23	1.02 ± 0.34	0.708
	6 months	0.95 ± 0.32	0.86 ± 0.23 *	0.318
E flow DT (ms)	Baseline	234 ± 50	214 ± 37	0.135
	6 months	230 ± 56	230 ± 53	0.958
IVRT (ms)	Baseline	99 ± 21	100 ± 30	0.891
	6 months	92 ± 17	95 ± 20	0.629
Mitral Ea (cm/s)	Baseline	7.7 ± 1.5	8.0 ± 2.2	0.583
	6 months	7.6 ± 2.4	9.0 ± 2.4 *	0.063
Mitral E/Ea	Baseline	9.7 ± 1.1	10.6 ± 2.7	0.158
	6 months	10.4 ± 3.5	8.7 ± 3.7 *	0.096

BP, blood pressure; DT, deceleration time; Ea, annular early diastolic velocity; Ht, height; IVRT, isovolumic relaxation time; IVS, interventricular septal wall thickness; LA, left atrial; LAVI, left atrial volume index; LVEDD, left ventricular end-diastole dimension; LVEF, left ventricular ejection fraction; LVESD, left ventricular end-systole dimension; LVM, left ventricular mass; NT-proBNP, N-terminal pro–brain natriuretic peptide; PW, posterior wall thickness; QOL, quality of life; RWT, relative wall thickness. * *p* < 0.05, baseline vs. 6-months by paired *t*-test.

**Table 3:** Comparisons of Contractile Motion at baseline and 6 months.

		Control (*n* = 24)	Spironolactone (*n* = 25)	*p*-Value
HR (1/s)	66 ± 13	68 ± 12	0.640	0.676
	68 ± 14	72 ± 15	0.963	0.287
Post-exercise HR (1/s)	Baseline	118 ± 16	115 ± 18	0.640
	6 months	122 ± 18	122 ± 20	0.963
Mean Sm (cm/s)	Baseline	7.0 ± 0.9	7.1 ± 1.2	0.628
	6 months	7.1 ± 0.8	7.2 ± 1.3	0.627
Post-exercise mean Sm (cm/s)	Baseline	8.3 ± 1.1	8.6 ± 1.5	0.410
	6 months	8.3 ± 1.0	9.2 ± 1.6 *	0.021
Post-exercise % increase of septal Sm	Baseline	33 ± 28	30 ± 32	0.757
	6 months	29 ± 20	29 ± 24	0.956
Post-exercise % increase of lateral Sm	Baseline	27 ± 28	30 ± 19	0.568
	6 months	21 ± 21	44 ± 30 *	0.003

HR, heart rate; Sm, systolic myocardial velocity. * *p* < 0.05, baseline vs. 6-months by paired *t*-test.

## Data Availability

Not applicable.
